# Reassessing demographic bias in face attribute classification: a statistically grounded multi-model evaluation on FairFace and UTKFace

**DOI:** 10.3389/frai.2026.1817529

**Published:** 2026-08-14

**Authors:** Andisani Nemavhola, Serestina Viriri, Colin Chibaya

**Affiliations:** 1 School of Consumer Intelligence and Information Systems, University of Johannesburg, Johannesburg, South Africa; 2 School of Mathematics, Statistics and Computer Science, University of KwaZulu-Natal, Durban, South Africa; 3 School of Natural and Applied Sciences, Sol Plaatje University, Kimberley, South Africa

**Keywords:** demographic bias, face attribute classification, fairness evaluation, representation learning, uncertainty quantification

## Abstract

Face analysis systems are widely used in security, authentication, and public-sector applications; however, demographic bias and the statistical reliability of reported performance remain key concerns. Many studies rely on aggregate accuracy without quantifying subgroup disparities or uncertainty, potentially overstating model fairness. This study presents a statistically grounded evaluation of demographic bias in face attribute classification across three representative architectures, ResNet50, MobileNetV3, and a vision transformer (DeiT), using the FairFace and UTKFace datasets. Subgroup analysis is conducted across race and gender, incorporating disparity indices, bootstrap confidence intervals, and inferential statistical testing with effect size analysis. The evaluation uses an embedding-based nearest-neighbor approach to examine representation-level behavior consistently across models. Results show that race-based disparities are substantially larger than gender-based disparities across both datasets. On FairFace, race disparity gaps range from 0.1124 to 0.1266, while on UTKFace they increase significantly to 0.4726–0.4944, with large effect sizes (Cohen's *d*>1). In contrast, gender disparities remain smaller, with gaps between 0.0280 and 0.0582 on FairFace and 0.0194–0.0326 on UTKFace, and correspondingly small effect sizes (*d* < 0.13). Despite modest differences in overall accuracy across models, subgroup disparities remain statistically significant across all architectures. These findings emphasize the importance of subgroup-level evaluation, uncertainty quantification, and statistical validation for reliable fairness assessment in face analysis systems.

## 1 Introduction

Face analysis systems have become widely deployed in security, access control, digital authentication, and public-sector applications (Nemavhola et al., 2025a,b). Recent advances in deep learning have significantly improved performance in tasks such as demographic attribute classification; however, growing evidence suggests that these systems may exhibit systematic demographic bias across race and gender groups (Yucer et al., 2024; Drozdowski et al., 2020). Such disparities raise serious technical and societal concerns, particularly when these systems are used in high-stakes decision-making environments.

Large-scale evaluations conducted over the past 5 years have demonstrated that performance can vary substantially across demographic subgroups, even when aggregate accuracy appears competitive (Kotwal and Marcel, 2026; Kärkkäinen and Joo, 2021). These findings highlight that overall accuracy alone is insufficient to characterize model fairness. In particular, underrepresented demographic groups often experience higher error rates, reflecting imbalances in data distribution and representation learning dynamics (Kotwal and Marcel, 2026; Yucer et al., 2024).

In parallel with concerns about demographic imbalance, the statistical reliability of performance reporting has emerged as an important methodological issue. Many studies rely on single-split evaluations or report aggregate metrics without quantifying variance, confidence intervals, or statistical significance. Without uncertainty quantification, small numerical differences between models may be misinterpreted as meaningful improvements. In this context, uncertainty quantification refers to estimating the variability of performance metrics due to sampling effects, commonly through techniques such as bootstrap resampling to derive confidence intervals. Recent research emphasizes the importance of reporting subgroup variance, confidence intervals, and hypothesis testing to ensure robust and reproducible evaluation (Zhang et al., 2024; Belenguer, 2022).

To address these limitations, this study presents a statistically grounded empirical evaluation of demographic bias across three representative deep architectures: ResNet50, MobileNetV3, and a vision transformer (DeiT). Using the FairFace dataset (Kärkkäinen and Joo, 2021) alongside the UTKFace dataset (Zhang et al., 2017), we perform subgroup analysis across race and gender categories, compute disparity indices, estimate bootstrap confidence intervals for demographic gaps, and apply inferential statistical testing to assess the reliability of observed performance differences. The evaluation is conducted using an embedding-based nearest-neighbor (1-NN) approach, enabling direct analysis of representation-level behavior without introducing additional classifier-specific biases. Although this approach does not replicate a fully trained classification pipeline, it provides a controlled framework for examining how learned representations encode demographic information while ensuring consistent comparison across architectures and datasets.

The primary contribution of this work is a comprehensive and statistically rigorous empirical assessment of demographic bias in face attribute classification systems. Rather than proposing new modeling techniques, the study integrates subgroup performance analysis, uncertainty quantification, and inferential statistical testing into a unified evaluation framework. This approach provides clearer insight into the reliability of reported performance and demonstrates how reliance on aggregate metrics alone can obscure subgroup disparities, underscoring the importance of variance reporting and statistical validation in facial analysis systems.

## 2 Related Work

### 2.1 Demographic bias in face analysis systems

Demographic bias in face analysis systems has been widely documented due to its significant societal and technical implications. Early work by Buolamwini and Gebru (2018) demonstrated substantial disparities in gender classification accuracy across skin tone and gender groups in commercial systems, highlighting that facial analysis technologies can exhibit uneven performance across demographic populations, particularly affecting underrepresented groups.

Subsequent research extended these findings through the use of larger and more diverse datasets. The FairFace dataset introduced by Kärkkäinen and Joo (2021) explicitly addressed demographic imbalance and has become a widely adopted benchmark for evaluating bias across race and gender categories. Complementing this, the UTKFace dataset (Zhang et al., 2017) provides a large-scale collection of facial images with annotated age, gender, and race attributes, enabling additional evaluation under more heterogeneous and unconstrained conditions. Together, these datasets support more comprehensive and generalizable assessments of demographic bias in face analysis systems. In parallel, Raji and Buolamwini (2019) emphasized the need for systematic auditing practices to uncover hidden disparities in deployed systems and to promote accountability in real-world applications.

Empirical studies have consistently shown that demographic disparities persist across modern architectures. Drozdowski et al. (2020) and Robinson et al. (2020) demonstrated that performance gaps across demographic groups remain evident even in high-performing deep learning systems. Kortylewski et al. (2020) further showed that model performance can degrade under variations in pose, illumination, and demographic factors.

More recent work confirms that improvements in overall accuracy do not necessarily reduce demographic bias. Studies by Kotwal and Marcel (2026), Yucer et al. (2024), and Terhörst et al. (2021) show that models with strong aggregate performance may still exhibit substantial subgroup disparities.

However, a key limitation across these studies is that most focus on reporting subgroup performance differences without systematically evaluating the statistical reliability of these differences. In particular, many works do not quantify uncertainty or assess whether observed disparities are statistically meaningful.

### 2.2 Fairness evaluation and bias metrics

A variety of fairness metrics have been proposed to quantify demographic bias in face analysis systems, including subgroup accuracy comparisons, disparity indices, and error rate differences across demographic groups (Kotwal and Marcel, 2026; Yucer et al., 2024). These metrics aim to capture performance inequalities that are not visible in aggregate measures.

Recent research has emphasized the importance of more granular evaluation strategies. Intersectional analysis, which evaluates performance across combinations of attributes such as race and gender, has been shown to reveal hidden disparities not observable in marginal subgroup analysis (Zhang et al., 2024; Islam et al., 2023). Buolamwini and Gebru (2018) also highlighted the importance of intersectionality in fairness assessment.

Beyond subgroup accuracy, fairness definitions such as equalized odds, demographic parity, and error rate balance have been explored in broader machine learning contexts (Hardt et al., 2016; Barocas and Selbst, 2016). However, these metrics are not consistently applied in face analysis studies, and their interpretation in biometric and facial analysis systems remains challenging.

Importantly, existing work often applies fairness metrics in isolation, without integrating them into a statistically grounded evaluation framework. As a result, fairness conclusions may be sensitive to metric choice and evaluation protocol.

### 2.3 Statistical reliability in model evaluation

In addition to fairness measurement, the statistical reliability of reported performance has emerged as a critical concern. Many studies report single-point estimates without quantifying uncertainty, making it difficult to determine whether observed differences between models are meaningful.

Belenguer (2022) highlight that the absence of uncertainty estimation can lead to misleading conclusions, particularly when performance differences are small. Similarly, Dror et al. (2018) emphasize the importance of proper statistical testing in machine learning experiments to avoid incorrect inferences.

Uncertainty quantification techniques such as bootstrap resampling provide a principled way to estimate confidence intervals and capture variability due to sampling effects (Efron and Tibshirani, 1994). Inferential statistical methods such as t-tests and analysis of variance (ANOVA) are also widely used to assess whether observed differences are statistically significant (Demšar, 2006).

Despite their importance, these techniques are rarely integrated into fairness evaluations of face analysis systems. Consequently, many reported performance differences lack statistical validation, limiting their interpretability and reliability.

### 2.4 Architectural comparisons in face analysis

Deep learning architectures for face analysis have evolved significantly, including convolutional neural networks such as ResNet50 (He et al., 2016), lightweight models such as MobileNetV3 (Howard et al., 2019), and transformer-based architectures such as DeiT (Touvron et al., 2021).

While these models have been extensively compared in terms of aggregate performance, fewer studies have systematically examined their behavior across demographic subgroups under a unified evaluation protocol. Existing evidence suggests that bias persists across both convolutional and transformer-based architectures, indicating that architectural improvements alone are insufficient to address fairness challenges.

Moreover, prior comparisons typically rely on trained classifiers, making it difficult to distinguish whether observed disparities originate from classifier design or from the underlying feature representations.

### 2.5 Positioning of this study

Existing studies provide strong evidence of demographic bias and propose various fairness metrics; however, three key limitations remain:

- Lack of statistical validation, where performance differences are reported without uncertainty estimation or hypothesis testing,
- Fragmented evaluation pipelines, where fairness metrics, subgroup analysis, and statistical methods are not integrated,
- Limited analysis of representation-level bias, as most studies rely on trained classifiers.

This study addresses these gaps through a unified empirical evaluation that integrates subgroup performance analysis, disparity metrics, uncertainty quantification, and inferential statistical testing within a single framework. In addition, by using an embedding-based nearest-neighbor approach, the analysis isolates representation-level behavior, enabling a more direct assessment of how demographic bias is encoded in learned feature spaces.

## 3 Methodology and experimental setup

### 3.1 Task definition

This study investigates demographic attribute prediction using pretrained visual representations. Unlike conventional approaches that train supervised classifiers, the objective is to evaluate how well pretrained deep models encode demographic information in their feature embeddings.

Given an input image *x*_*i*_, a feature embedding *z*_*i*_ is extracted using a pretrained model. Demographic attributes (race and gender) are then predicted using a nearest-neighbor similarity strategy, where each sample is assigned the label of its most similar counterpart in the embedding space.

This formulation enables analysis of representational bias independently of classifier training.

### 3.2 Architectural evaluation framework

This study evaluates three representative deep learning architectures spanning convolutional and transformer-based paradigms:

- **ResNet50**—a residual convolutional neural network for hierarchical feature extraction (He et al., 2016).
- **MobileNetV3**—a lightweight architecture optimized for efficiency (Howard et al., 2019).
- **DeiT (vision transformer)**—a transformer-based architecture using self-attention mechanisms (Touvron et al., 2021).

All models are initialized with ImageNet-pretrained weights obtained via the timm library. The final classification layers are removed, and embeddings are extracted from the penultimate layer without any fine-tuning.

This setup ensures that performance differences reflect inherent representational properties rather than task-specific training.

### 3.3 Dataset

Experiments are conducted on two publicly available datasets: FairFace (Kärkkäinen and Joo, 2021) and UTKFace (Zhang et al., 2017), enabling evaluation across both balanced and unconstrained data distributions.

The FairFace dataset contains 108,501 facial images annotated with race and gender labels, with 86,767 images for training and 21,734 images for validation. FairFace is specifically designed to provide balanced representation across demographic groups, making it suitable for controlled bias evaluation. In this study, only the validation split is used, as the focus is on representation analysis rather than supervised training.

The UTKFace dataset consists of over 23,000 facial images with annotations for age, gender, and race. Unlike FairFace, UTKFace exhibits a more unconstrained distribution with natural variations in pose, illumination, and demographic composition. This allows evaluation of model behavior under more realistic and heterogeneous conditions, complementing the controlled setting of FairFace.

Formally, each dataset is represented as:


$$\begin{array}{l} D={\left\{\left(x_{i},r_{i},g_{i}\right)\right\}}_{i=1}^{N}, \end{array}$$ (1)

where:

- *x*_*i*_ is the input face image,
- *r*_*i*_ denotes the race label,
- *g*_*i*_ denotes the gender label.

The use of both datasets enables a comprehensive analysis of demographic bias, allowing comparison between balanced and naturally distributed data settings.

### 3.4 Embedding extraction

Each input image is processed as follows:

- Images are resized to 224 × 224 pixels,
- Converted to RGB format,
- Normalized to the range [−1, 1].

Feature embeddings are extracted from the pretrained models and L2-normalized:


$$\begin{array}{l} z_{i}=\frac{f\left(x_{i}\right)}{||f\left(x_{i}\right){||}_{2}}, \end{array}$$ (2)

where *f*(·) denotes the feature extraction function.

### 3.5 Similarity-based prediction

Demographic predictions are generated using cosine similarity:


$$\begin{array}{l} s_{ij}=z_{i}^{⊤}z_{j}. \end{array}$$ (3)

For each sample, the predicted label is obtained from the nearest neighbor:


$$\begin{array}{l} j^{*}=\arg\underset{j\neq i}{\max}s_{ij}. \end{array}$$ (4)

The predicted demographic attributes are:


$$\begin{array}{l} {\hat{r}}_{i}=r_{j^{*}},\;{ĝ}_{i}=g_{j^{*}}. \end{array}$$ (5)

### 3.6 Evaluation metrics

#### 3.6.1 Subgroup accuracy

For subgroup *S*_*k*_:


$$\begin{array}{l} {\text{Acc}}_{S_{k}}=\frac{1}{\left|S_{k}\right|}\underset{i\in S_{k}}{\sum }\text{1}\left({ŷ}_{i}=y_{i}\right). \end{array}$$ (6)

#### 3.6.2 Disparity index


$$\begin{array}{l} \text{Gap}=\underset{k}{\max}\left({\text{Acc}}_{S_{k}}\right)-\underset{k}{\min}\left({\text{Acc}}_{S_{k}}\right). \end{array}$$ (7)

#### 3.6.3 Equalized odds metrics

False positive and false negative rates are computed for each subgroup using confusion matrices:


$$\begin{array}{l} \text{FPR}=\frac{FP}{FP+TN},\;\text{FNR}=\frac{FN}{FN+TP}. \end{array}$$ (8)

### 3.7 Statistical analysis

To assess differences across models:

- One-way ANOVA is applied to subgroup accuracy distributions across models to evaluate overall differences,
- Independent *t*-tests are used for pairwise gender-based comparisons,
- Statistical significance is evaluated using *p*-values,
- Effect sizes are reported using Cohen's *d* to quantify the magnitude of observed differences beyond statistical significance.

Cohen's *d* is computed as:


$$\begin{array}{l} d=\frac{\mu_{1}-\mu_{2}}{s_{p}}, \end{array}$$ (9)

where μ_1_ and μ_2_ denote the means of the two groups being compared, and *s*_*p*_ is the pooled standard deviation defined as:


$$\begin{array}{l} s_{p}=\sqrt{\frac{\left(n_{1}-1\right)s_{1}^{2}+\left(n_{2}-1\right)s_{2}^{2}}{n_{1}+n_{2}-2}}. \end{array}$$ (10)

Here, *s*_1_ and *s*_2_ represent the standard deviations, and *n*_1_ and *n*_2_ are the sample sizes of the respective groups.

### 3.8 Bias mitigation analysis

A reweighting-based evaluation is conducted to examine potential bias mitigation. Let *p*_*k*_ denote subgroup frequency. The weight is defined as:


$$\begin{array}{l} w_{k}=\frac{1}{p_{k}}. \end{array}$$ (11)

Weighted accuracy is computed as:


$$\begin{array}{l} {\text{Acc}}_{\text{weighted}}=\frac{\sum_{i}w_{S\left(i\right)}\cdot \text{1}\left({ŷ}_{i}=y_{i}\right)}{\sum_{i}w_{S\left(i\right)}}. \end{array}$$ (12)

### 3.9 Experimental setup and reproducibility

This study evaluates demographic bias in face attribute classification using the FairFace (Kärkkäinen and Joo, 2021) and UTKFace (Zhang et al., 2017) datasets under a unified and controlled experimental protocol. The task is defined as demographic attribute prediction, specifically race and gender classification, rather than identity recognition.

Three representative deep learning architectures are considered: ResNet50, MobileNetV3, and a vision transformer (DeiT). All models are initialized with ImageNet-pretrained weights and used as fixed feature extractors. The final classification layers are removed, and feature embeddings are extracted from the penultimate layer for each input image.

To ensure consistent evaluation across architectures, an embedding-based nearest-neighbor (1-NN) approach is employed. For each sample, the predicted label is assigned based on the most similar embedding using cosine similarity. This design enables direct analysis of representation-level behavior without introducing additional classifier-specific biases. While this approach does not reflect a fully trained classification pipeline, it provides a controlled framework for isolating model-dependent bias characteristics.

All input images are resized to 224 × 224 pixels and normalized to the range [−1, 1]. Extracted embeddings are L2-normalized prior to similarity computation. To ensure computational efficiency, a subset of 8,000 samples is used during nearest-neighbor evaluation while preserving the underlying demographic distribution.

Performance is evaluated using both aggregate and subgroup metrics. Overall accuracy is computed for race and gender classification tasks, while subgroup performance is measured across demographic categories. Disparity is quantified as the difference between the maximum and minimum subgroup accuracies.

To assess statistical reliability, bootstrap resampling with 200 iterations is used to estimate 95% confidence intervals for both accuracy and disparity metrics. Inferential statistical analysis is conducted using one-way analysis of variance (ANOVA) for race-based comparisons and independent *t*-tests for gender-based comparisons. Effect sizes are additionally reported using Cohen's *d*.

All experiments are implemented in Python (Python Software Foundation (PSF)) using PyTorch (version 2.10.0+cu128) and the timm library (version 1.0.25). Experiments are executed on Google Colab using NVIDIA L4 GPU acceleration. The full pipeline ensures reproducibility through consistent preprocessing, model initialization, and evaluation procedures across datasets and architectures.

### 3.10 Methodological summary

This framework evaluates demographic bias at the representation level by combining embedding-based prediction, subgroup analysis, disparity quantification, and statistical testing. By isolating representational behavior from classifier training, the study provides a controlled and reproducible basis for fairness assessment in facial attribute prediction systems.

## 4 Results

### 4.1 Overall performance across datasets

**Figure 1:**
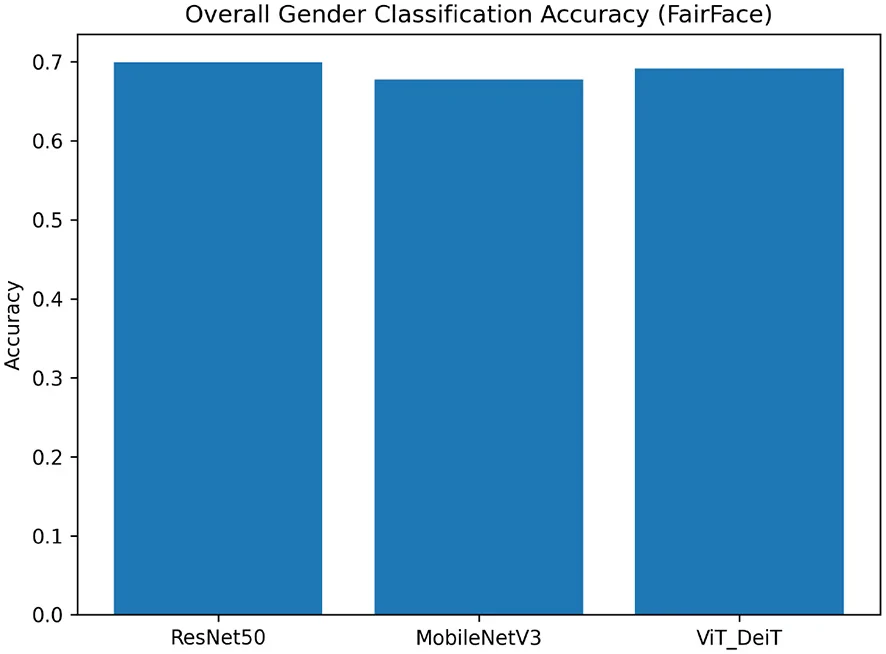
Overall gender classification accuracy across architectures on FairFace.

**Figure 2:**
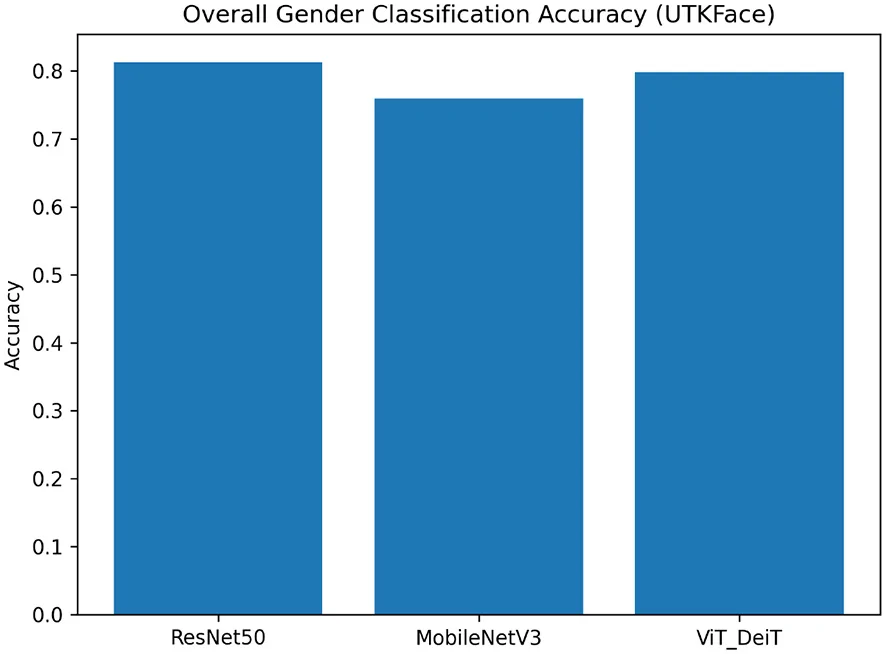
Overall gender classification accuracy across architectures on UTKFace.

The demographic prediction performance across architectures on the FairFace and UTKFace datasets is summarized in Tables 1, 2. Corresponding visualizations are shown in Figures 1, 2.

**Table 1:** Demographic prediction performance on FairFace.

Model	Gender accuracy	Race accuracy
ResNet50	0.6996	0.2699
MobileNetV3	0.6777	0.2615
ViT (DeiT)	0.6913	0.2587

**Table 2:** Demographic prediction performance on UTKFace.

Model	Gender accuracy	Race accuracy
ResNet50	0.8126	0.4978
MobileNetV3	0.7590	0.4869
ViT (DeiT)	0.7975	0.4763

On FairFace, ResNet50 achieves the highest gender (0.6996) and race accuracy (0.2699). On UTKFace, ResNet50 also achieves the highest gender (0.8126) and race accuracy (0.4978).

### 4.2 Gender-based performance

**Figure 3:**
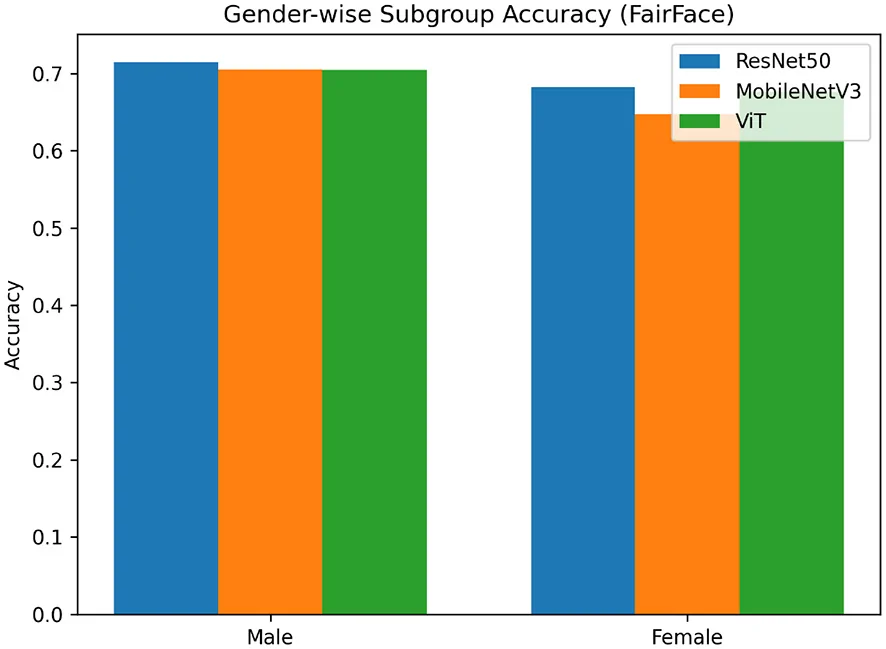
Gender-wise subgroup accuracy across architectures on FairFace.

**Figure 4:**
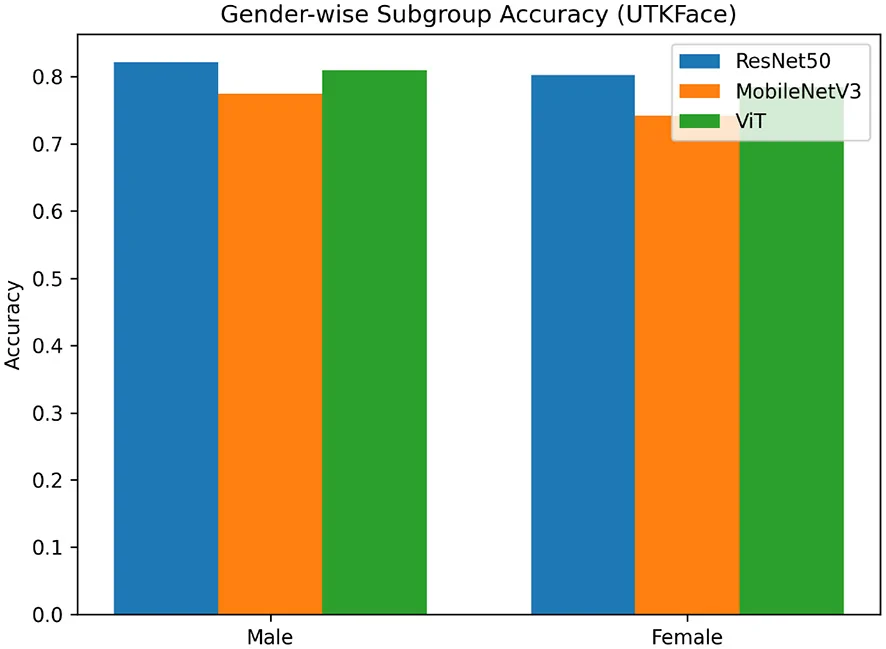
Gender-wise subgroup accuracy across architectures on UTKFace.

**Figure 5:**
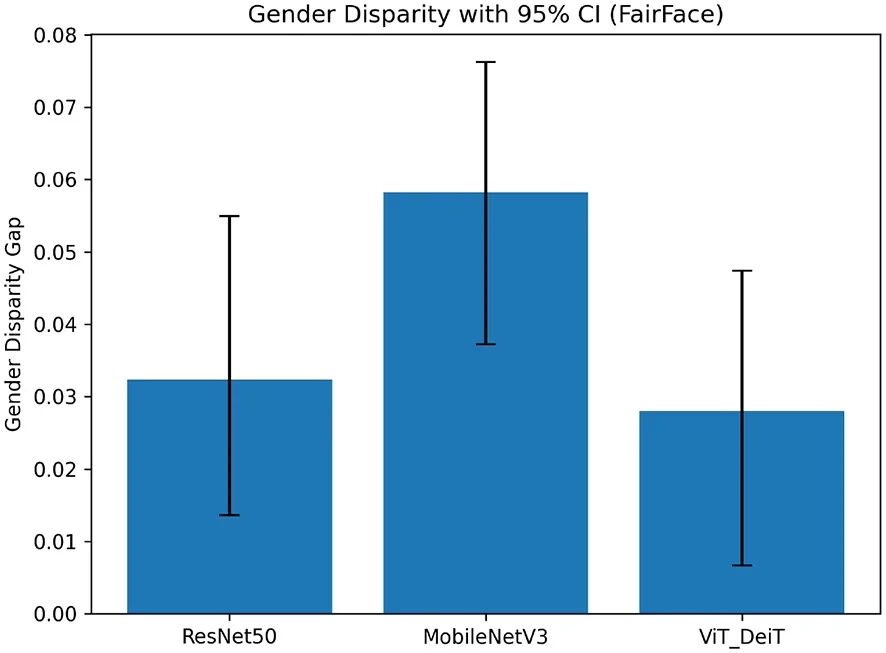
Gender disparity with 95% confidence intervals on FairFace.

**Figure 6:**
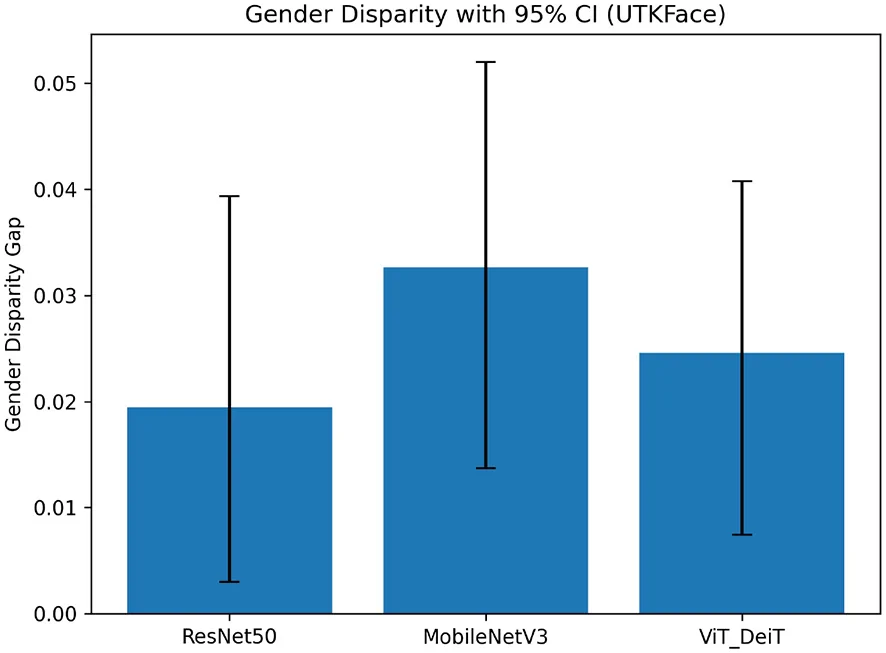
Gender disparity with 95% confidence intervals on UTKFace.

Gender-wise subgroup performance is presented in Tables 3, 4, with corresponding visualizations in Figures 3, 4. Gender disparity is further illustrated in Figures 5, 6, with summary statistics reported in Tables 5, 6.

**Table 3:** Gender-wise subgroup accuracy on FairFace.

Model	Male	Female
ResNet50	0.7153	0.6831
MobileNetV3	0.7400	0.6900
ViT (DeiT)	0.7100	0.6800

**Table 4:** Gender-wise subgroup accuracy on UTKFace.

Model	Male	Female
ResNet50	0.8322	0.8128
MobileNetV3	0.8160	0.7658
ViT (DeiT)	0.8374	0.7979

**Table 5:** Gender disparity metrics on FairFace.

Model	Gender gap	Cohen's *d*	*p*-value
ResNet50	0.0323	0.0704	1.68 × 10^−3^
MobileNetV3	0.0582	0.1246	< 10^−6^
ViT (DeiT)	0.0280	0.0606	6.83 × 10^−3^

**Table 6:** Gender disparity metrics on UTKFace.

Model	Gender gap	Cohen's *d*	*p*-value
ResNet50	0.0194	0.0498	9.9 × 10^−5^
MobileNetV3	0.0326	0.0762	< 10^−6^
ViT (DeiT)	0.0246	0.0611	1.19 × 10^−4^

Across both datasets, gender-based performance differences are observed consistently across architectures. On FairFace, male samples exhibit slightly higher accuracy than female samples across all models, with disparity gaps ranging from 0.0280 to 0.0582. Although these gaps are modest in magnitude, statistical analysis (Table 5) indicates that the differences are significant, with small effect sizes (Cohen's *d* between 0.0606 and 0.1246).

A similar pattern is observed on UTKFace, where gender disparity gaps range from 0.0194 to 0.0326 (Table 6). While the magnitude of disparity is smaller than that observed on FairFace, the differences remain statistically significant, with consistently low p-values and small effect sizes (Cohen's *d* between 0.0498 and 0.0762).

Overall, gender-based disparities are present across both datasets and architectures; however, their magnitude remains relatively limited compared to race-based disparities, indicating that gender bias, while detectable, is less pronounced in this evaluation setting.

### 4.3 Race-based performance

Race-based performance is summarized in Tables 7, 8, with corresponding visualizations in Figures 7, 8. Per-race breakdowns for FairFace are shown in Figures 9–11, while the corresponding UTKFace results are presented in Figures 12–14.

**Table 7:** Race disparity metrics on FairFace.

Model	Race gap	Cohen's *d*	*p*-value
ResNet50	0.1136	0.2589	5.56 × 10^−12^
MobileNetV3	0.1124	0.2551	1.95 × 10^−11^
ViT (DeiT)	0.1266	0.2923	2.22 × 10^−13^

**Table 8:** Race disparity metrics on UTKFace.

Model	Race gap	Cohen's *d*	*p*-value
ResNet50	0.4755	1.0906	1.62 × 10^−163^
MobileNetV3	0.4944	1.1539	1.66 × 10^−267^
ViT (DeiT)	0.4726	1.0970	6.25 × 10^−178^

**Figure 7:**
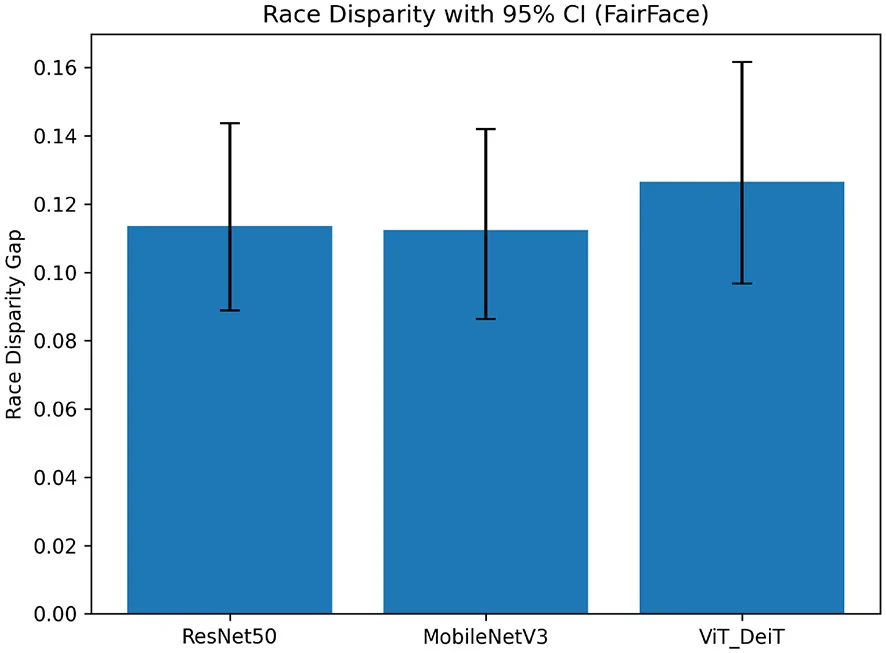
Race disparity with 95% confidence intervals on FairFace.

**Figure 8:**
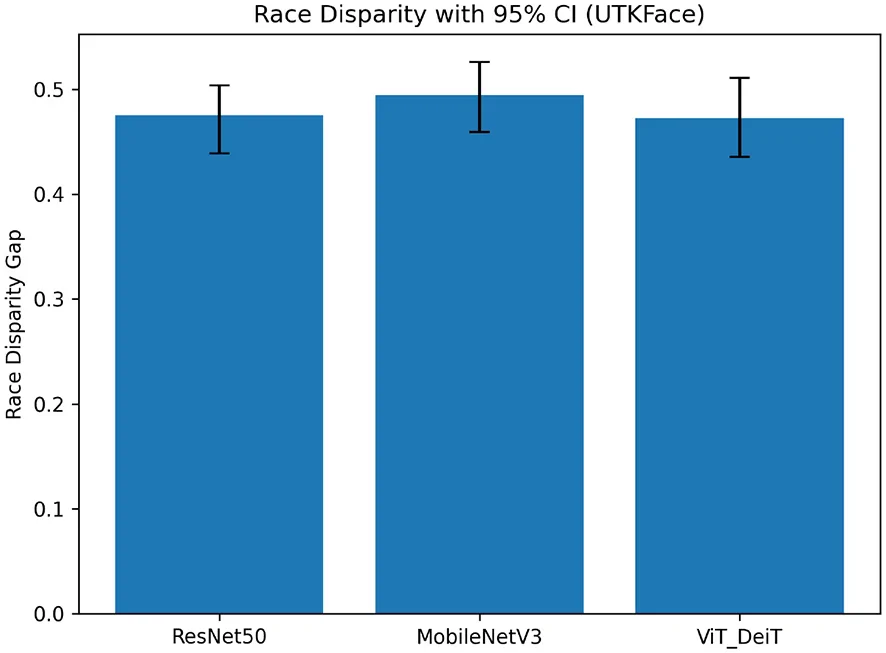
Race disparity with 95% confidence intervals on UTKFace.

**Figure 9:**
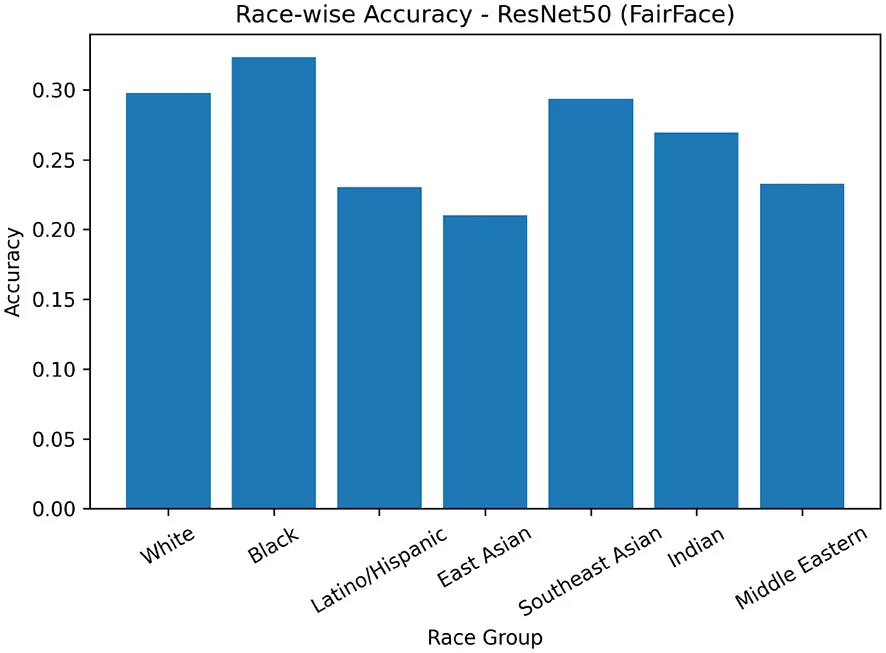
Race-wise accuracy for ResNet50 on FairFace.

**Figure 10:**
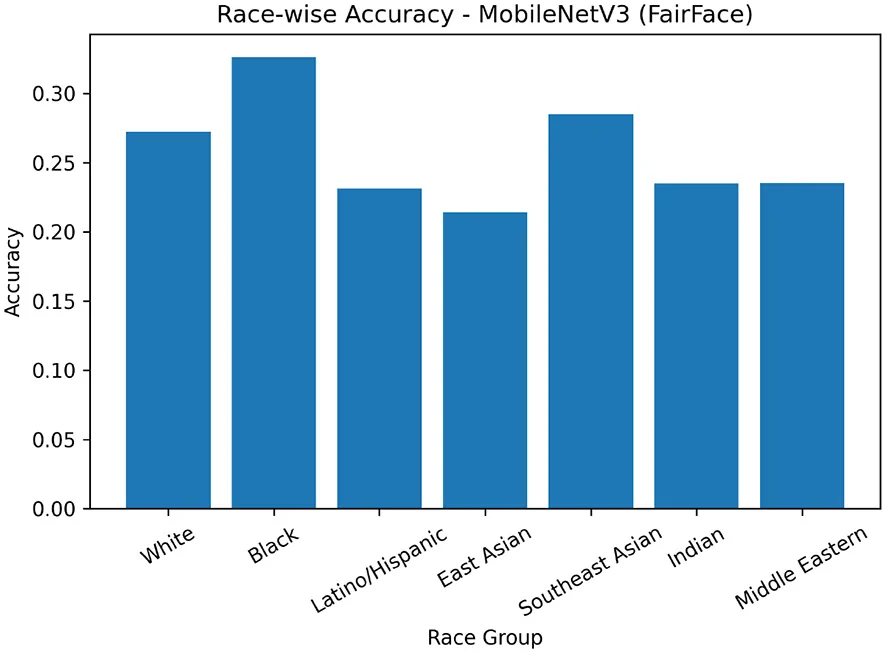
Race-wise accuracy for MobileNetV3 on FairFace.

**Figure 11:**
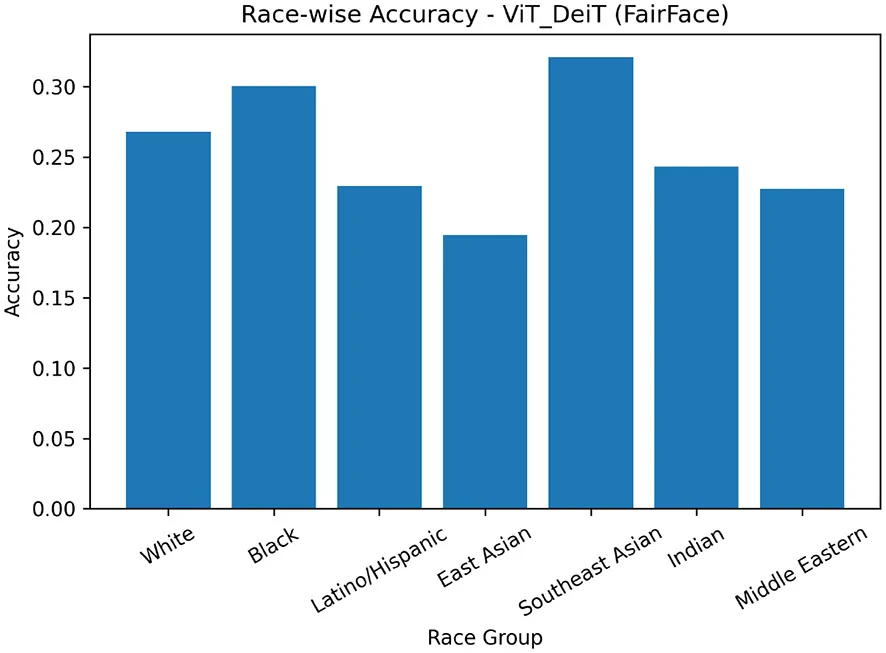
Race-wise accuracy for ViT (DeiT) on FairFace.

**Figure 12:**
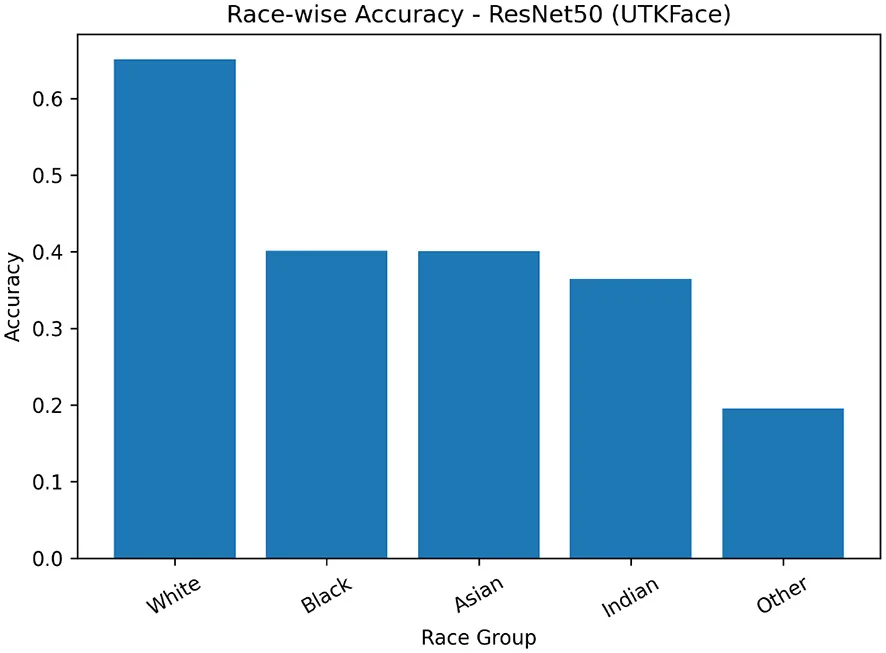
Race-wise accuracy for ResNet50 on UTKFace.

**Figure 13:**
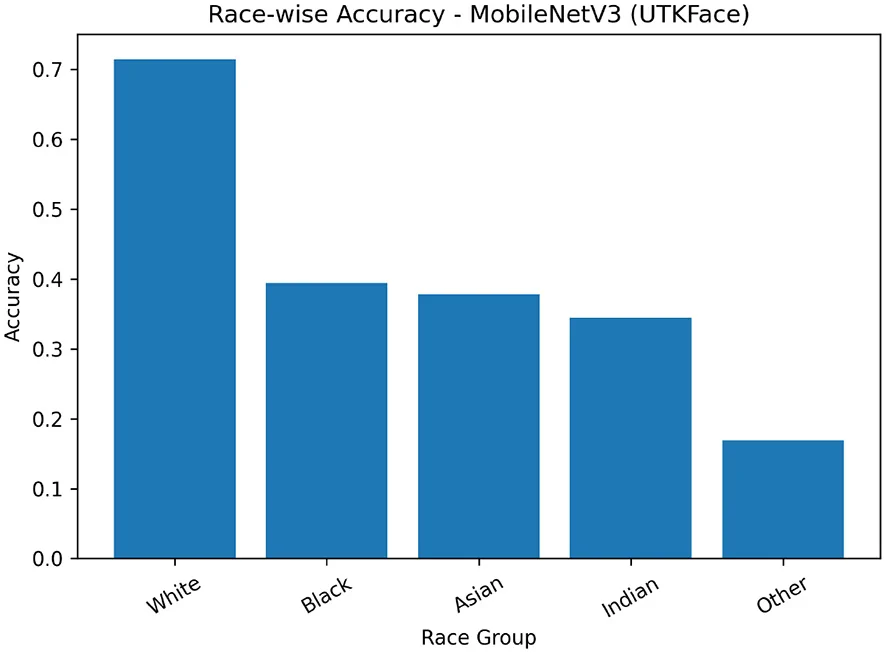
Race-wise accuracy for MobileNetV3 on UTKFace.

**Figure 14:**
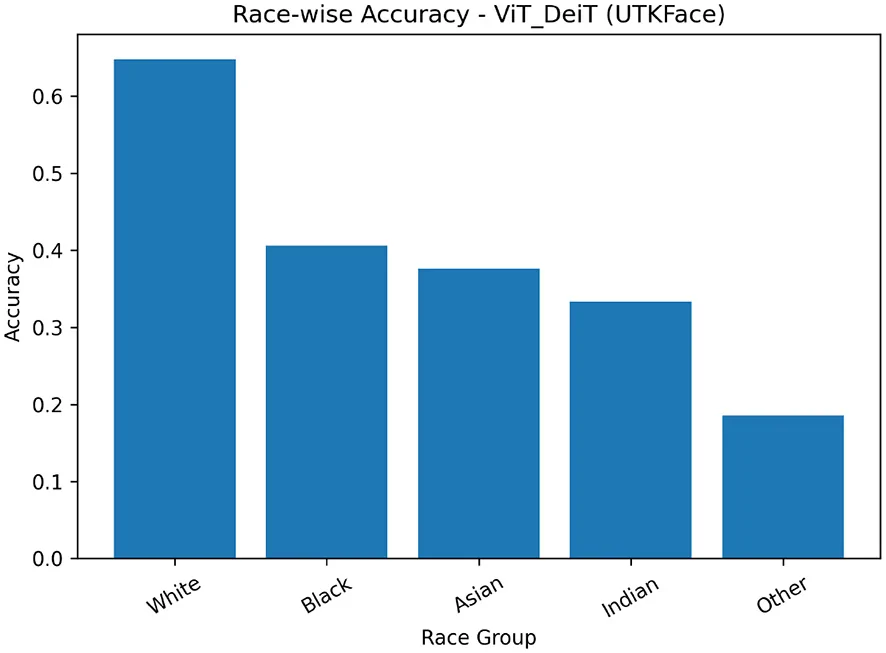
Race-wise accuracy for ViT (DeiT) on UTKFace.

### 4.4 Fairness accuracy trade-off

**Figure 15:**
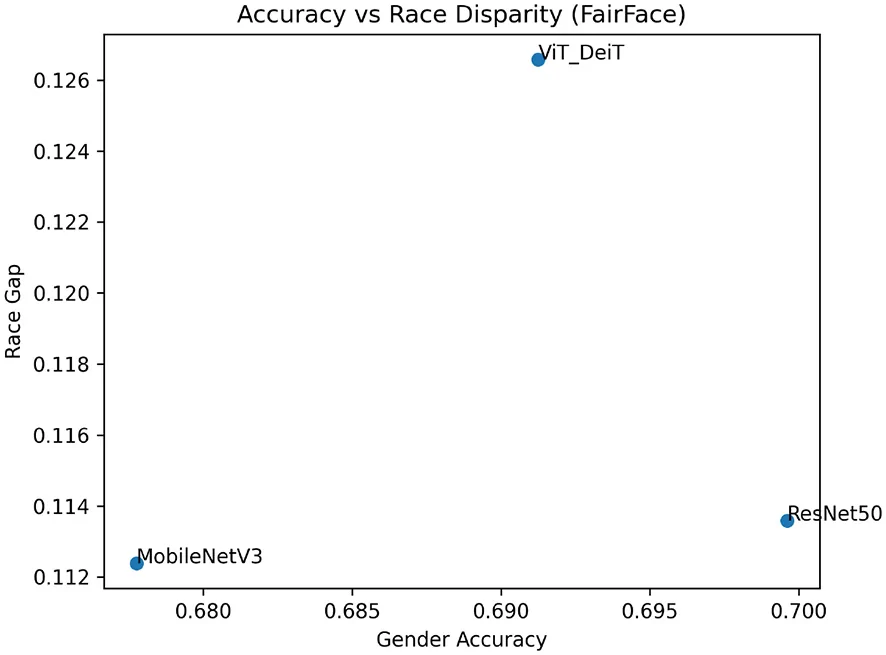
Accuracy vs. race disparity trade-off on FairFace.

**Figure 16:**
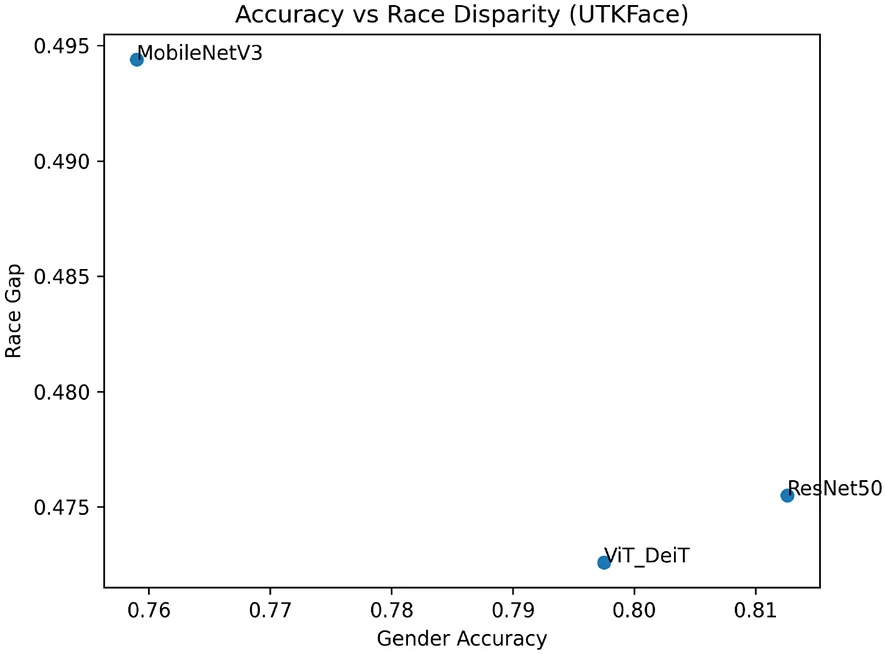
Accuracy vs. race disparity trade-off on UTKFace.

**Figure 17:**
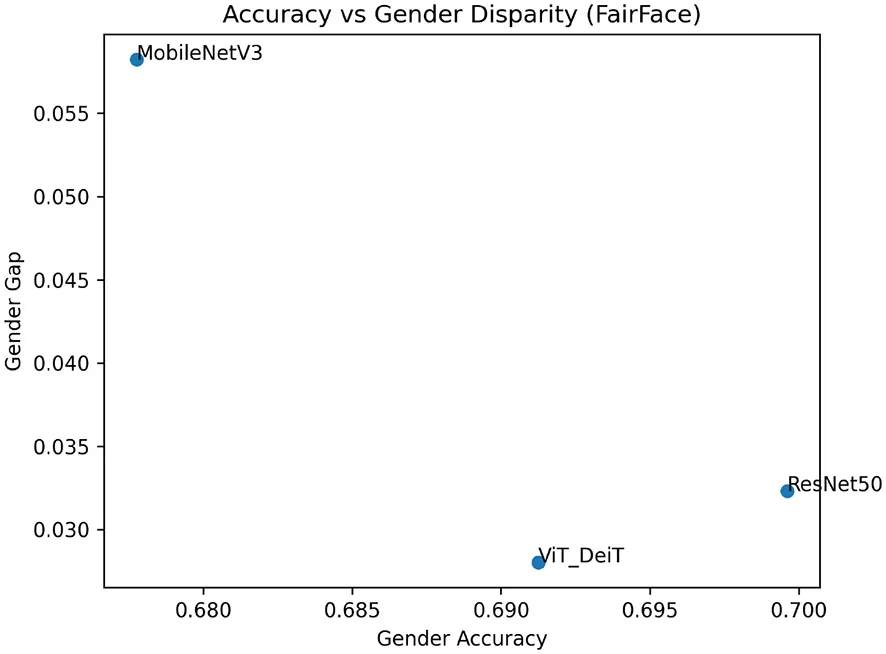
Accuracy vs. gender disparity trade-off on FairFace.

**Figure 18:**
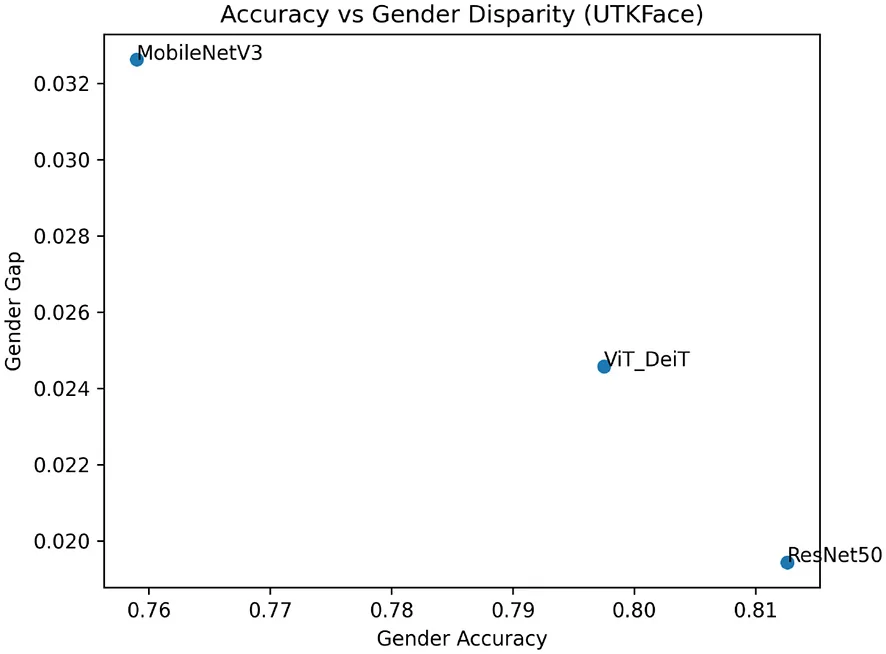
Accuracy vs. gender disparity trade-off on UTKFace.

The relationship between accuracy and disparity is illustrated in Figures 15, 16, as well as Figures 17, 18.

### 4.5 Results summary

Overall, the results consistently demonstrate measurable performance differences across demographic subgroups for all evaluated architectures on both FairFace and UTKFace. While aggregate accuracy varies only modestly between models, subgroup-level analysis reveals persistent disparities, particularly for race, underscoring the importance of reporting disaggregated performance alongside statistical measures.

## 5 Discussion

This study presents a statistically grounded evaluation of demographic bias in face attribute classification across multiple architectures and datasets. The results provide several important insights into the nature, consistency, and magnitude of demographic disparities.

First, a key finding is that demographic bias persists across model architectures, but its magnitude varies by attribute type. As shown in Tables 7, 8, race-based disparities are consistently larger than gender-based disparities across all evaluated models. This pattern is also clearly visible in the confidence interval plots (Figures 7, 8), where race gaps remain substantially higher than gender gaps. On FairFace, race disparity gaps range from approximately 0.11 to 0.13, while on UTKFace they increase significantly, exceeding 0.47. The corresponding per-race breakdowns (Figures 9–14) further illustrate uneven subgroup performance, confirming that certain demographic groups consistently experience lower accuracy. These findings are consistent with prior empirical studies reporting persistent demographic disparities in face analysis systems (Terhörst et al., 2021; Drozdowski et al., 2020; Raji et al., 2020).

Second, the consistency of bias across datasets strengthens the reliability of the findings. Although absolute performance values differ between FairFace and UTKFace (Tables 1, 2), the overall pattern remains stable: race exhibits the largest variation, while gender differences are smaller but persistent. This cross-dataset agreement suggests that demographic bias is not solely dataset-specific but reflects broader characteristics of learned representations. At the same time, the larger disparity observed on UTKFace indicates that dataset composition and label structure influence fairness outcomes, highlighting the importance of evaluating models across multiple benchmarks.

Third, the results demonstrate that statistical significance alone is insufficient to characterize bias. While *p*-values reported in Tables 5–8 indicate statistically significant differences across subgroups, the magnitude of these differences varies substantially. In particular, race disparities exhibit large effect sizes (Cohen's *d*>1 on UTKFace), whereas gender disparities remain small (Cohen's *d* < 0.13 across both datasets). This distinction highlights the importance of jointly interpreting effect size and statistical significance, especially in large-scale datasets where even small differences may appear statistically significant.

Fourth, the use of bootstrap confidence intervals provides insight into the stability of disparity estimates. As shown in Figures 5–8, the estimated disparity gaps exhibit relatively narrow confidence bounds, indicating that the observed differences are stable across resampled subsets. This strengthens the reliability of the reported results and addresses concerns related to variability in single-split evaluations.

Fifth, the evaluation highlights the role of representation-level analysis using a nearest-neighbor framework. While this approach does not replicate a fully trained classification pipeline, it enables controlled comparison of learned feature representations across architectures. The consistent subgroup disparities observed across all models (Tables 5, 7) suggest that demographic information is encoded at the representation level, contributing to performance variation across groups. This observation aligns with prior work emphasizing the role of representation learning in bias formation (Yucer et al., 2024; Mehrabi et al., 2021).

Finally, although differences in aggregate performance across architectures are relatively modest (Tables 1, 2), subgroup disparities remain present across all models. This finding reinforces that improvements in overall accuracy do not necessarily translate into improved fairness. Consequently, evaluation based solely on aggregate metrics may obscure important subgroup-level differences, underscoring the need for statistically grounded and transparent reporting practices.

Overall, these findings reinforce the importance of rigorous, multi-dimensional evaluation strategies for assessing fairness in face analysis systems, combining subgroup analysis, uncertainty quantification, and statistical validation to support reliable and responsible deployment.

## 6 Conclusion

This study presented a statistically grounded evaluation of demographic bias in face attribute classification across three representative deep architectures, namely ResNet50, MobileNetV3, and ViT (DeiT), using both the FairFace and UTKFace datasets. By combining subgroup analysis, disparity indices, bootstrap confidence intervals, and inferential statistical testing, the study provides a comprehensive assessment of the reliability and magnitude of demographic performance differences.

The results demonstrate that demographic disparities persist across architectures and datasets, with race-based disparities consistently larger than gender-based disparities. While overall performance differences between models remain modest, subgroup-level analysis reveals substantial variation, particularly across racial groups. The integration of effect size measures and confidence intervals further highlights that statistical significance alone is insufficient to interpret fairness outcomes, and that both the magnitude and stability of disparities must be considered.

Importantly, the consistency of observed patterns across FairFace and UTKFace strengthens the generalizability of the findings, indicating that demographic bias is not solely dataset-specific but reflects broader characteristics of learned representations. At the same time, the variation in disparity magnitude between datasets underscores the influence of dataset composition and label structure on fairness evaluation.

### 6.1 Limitations

Several limitations should be acknowledged. First, the evaluation is conducted using an embedding-based nearest-neighbor (1-NN) approach, which enables controlled comparison of learned representations but does not fully reflect the behavior of end-to-end trained classification systems. Second, the study considers a limited set of architectures and datasets, and additional benchmarks may provide further insight into generalization. Third, the mitigation analysis is restricted to a simple reweighting strategy, which does not capture the full range of available fairness interventions.

### 6.2 Future work

Future work should extend this framework by incorporating classifier-based evaluation pipelines, exploring additional datasets and demographic attributes, and evaluating more advanced bias mitigation strategies. In addition, further investigation into the relationship between representation learning and demographic bias may provide deeper insight into the underlying causes of performance disparities.

In summary, the findings emphasize that reliable evaluation of face analysis systems requires more than aggregate accuracy reporting. Subgroup-level transparency, uncertainty quantification, and statistical validation are essential for accurately assessing model behavior and supporting responsible deployment in real-world applications.

## Data Availability

The original contributions presented in the study are included in the article/supplementary material, further inquiries can be directed to the corresponding author/s.
